# Effects of Cavity Thickness on the Replication of Micro Injection Molded Parts with Microstructure Array

**DOI:** 10.3390/polym14245471

**Published:** 2022-12-14

**Authors:** Shaofei Jiang, Yuansong Zhang, Haowei Ma, Xiaoqiang Zha, Xiang Peng, Jiquan Li, Chunfu Lu

**Affiliations:** 1 College of Mechanical Engineering, Zhejiang University of Technology, Hangzhou 310014, China; 2 Taizhou Key Laboratory of Advanced Manufacturing Technology, Taizhou 318014, China; 3 Industrial Design and Research Institute, Zhejiang University of Technology, Hangzhou 310014, China

**Keywords:** uniformity, micro-injection molding, cavity thickness, microstructure array

## Abstract

Parts with microstructure arrays have been widely used in biotechnologies and optical technologies, and their performances are affected by replication uniformity. The uniformity of the microstructure is still a challenge in micro-injection molded parts and is greatly affected by the cavity thickness and process parameters. In this study, the replication uniformity of microstructures is experimentally investigated. The relationship between the replication uniformity and cavity thickness was explored through single-factor experiments. Additionally, the impacts of the process parameters on the replication uniformity were also studied through uniform design experiments. A regression equation was established to describe the quantitative relationship between the important parameters and replication uniformity. The results showed that the replication uniformity of microstructures increases by 39.82% between the cavity with the thickness of 0.5 mm and a cavity of 0.7 mm. In addition, holding time is the most significant factor influencing the replication uniformity, followed by mold temperature, melt temperature, and injection speed. It is concluded that the thickness of cavity and the process parameters have significant influence on the replication uniformity. The experimental results provide important data on how to improve the replication uniformity of parts with microstructure arrays.

## 1. Introduction

Parts with microstructure arrays have been widely used in biotechnologies and optical technologies and are the subject of significant demand [1,2,3,4]. Polymers are widely used in various fields, including the manufacture of parts with a microstructures array [5,6]. There are several methods for manufacturing polymer parts with microstructure arrays [7,8,9,10]. Micro injection molding (μIM) is the preferred manufacturing method because of its capability for mass-production and its low production cost [11,12,13]. However, the uneven pressure and temperature distribution during μIM usually introduces non-uniform microstructures in the molded parts, especially for the microstructures with different distances from the gate [14,15].

The replication uniformity will significantly influence the performances of parts with microstructure arrays [16,17,18,19]. To further improve the replication uniformity of parts, many researchers have studied the influence of the pressure and temperature distribution on microstructure replication of the molded parts [20,21,22]. There are many factors affecting temperature and pressure distribution [15,23,24]. The cavity thickness and process parameters will affect the melt temperature and cavity pressure distribution, as well as the final replication uniformity of the microstructures [25,26].

Up to now, some researchers have reported the effects of cavity thickness on replication quality [26,27,28,29,30]. Valtteri Kalima et al. [29] reported that the thick cavity thickness showed better replication of the grooves than the thin one for both aspect ratios. Conversely, Davide Masato et al. [30] indicated that a small cavity thickness is more conducive to improving the replication quality and that a higher cavity thickness tends to limit the replication during the injection phase, due to a lower increase in cavity pressure. The effects of cavity thickness are not consistent among different studies reported in the literature. Furthermore, when considering the effects of cavity thickness on replication quality in micro-injection molding, the effects on uniformity are often overlooked. Therefore, it is important to investigate how cavity thickness influences replication uniformity to provide guidance for improving uniformity.

In the last few years, several researchers have focused on the influences of processing parameters to improve the replication uniformity of the injection-molded parts with microstructure arrays [16,17,20,31,32]. Lucchetta et al. [31] indicated that to maximize both the average values and uniformity of the microfeatures, the process parameters need to be performed at a low injection speed and a high holding pressure. Matschuk et al. [32] explored the arrays of 40 nm wide pillars and optimized process parameters to enhance the replication quality that is described by the height, width, and uniformity of the nanoscopic features. Song et al. [17] investigated the effect of process parameters on the uniformity of porous arrays. However, the existing research on the influence of process parameters on the uniformity of rectangular microstructure arrays is not comprehensive. For this reason, adjusting the process parameters of the molding process to improve the uniformity of the part is an unexplored area of study.

This study discussed the effects of cavity thickness and process parameters on the replication depth and uniformity of microstructures. The relationship between the replication uniformity and cavity thickness was investigated by single-factor experiments. In addition, uniform design experiments were adopted to examine the effects of five process parameters on replication depth and replication uniformity of microstructures. After experiments, the cavity thickness and process parameters were analyzed to find out which of the major parameters influence the replication uniformity of the microstructure array. The results will contribute to a better understanding of replication quality for the microstructure array.

## 2. Materials and Methods

### 2.1. Experiment Material

**Figure 1:**
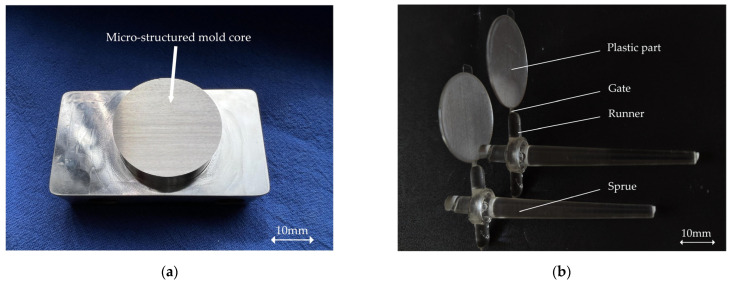
Photographs of the micro-structured mold core and parts: (**a**) insert with microstructure array; (**b**) injection-molded sample.

Figure 1a shows the disk-shaped mold insert with a diameter of 30 mm, on which rectangular microchannels with a width of 200 μm and depth of 110 μm are evenly distributed and oriented horizontally to the melt-flow direction. In addition, the spacing of the array is set as 100 μm. The microstructure arrays on the insert were manufactured by micro-grinding. Plastic parts with microstructure arrays were molded by μIM, as shown in Figure 1b.

**Figure 2:**
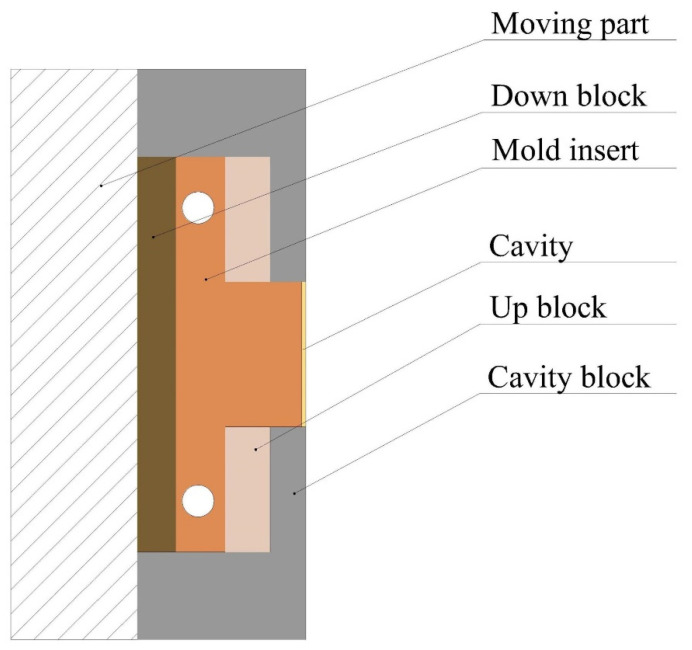
Schematic diagram of the mold assembly.

To investigate the effect of cavity thickness on the microstructure uniformity of the plastic part, a set of molds with removable inserts and adjustable cavity thickness were designed as illustrated in Figure 2.

In addition, only one cavity was designed, to avoid unbalanced filling. The mold insert was placed on the moving half. The replaceable inserts have the aim of minimizing mold manufacturing costs and increasing mold versatility [33], and they can eliminate the influence of the cavity surface. The cavity thickness was determined by the combination of up and down blocks, as shown in Figure 2. According to the team’s experience, when the cavity thickness is less than 0.3 mm, the molding process window is very narrow, which will limit the determination of the process parameter level in the experimental design. According to the existing research, combined with the geometric structure of plastic parts and the material characteristics of PMMA, three cavity thicknesses of 0.3 mm, 0.5 mm, and 0.7 mm were selected, as shown in Table 1 [15,28,34,35].

PMMA (HT55X, Sumitomo Chemical Co., Ltd., Tokyo, Japan) was selected due to the wide application of this material in the optical field. The experiments were implemented using an injection-molding machine (VE400(2)-80h-B, Ningbo Zhafir Plastics Machinery Manufacturing Co., Ltd., Ningbo, China). To control the mold temperature, a mold temperature machine (BTM-H, Shenzhen Borack Machinery Co., Ltd., Shenzhen, China) was used in the experiment, which can control the temperature error within a range of ±1 °C.

### 2.2. Experiment Design

The single variable method was carried out with changed cavity thickness to analyze the effects of cavity thickness on the uniformity of microstructure array. The uniform design focuses on the uniform distribution of test points within the test range to obtain the largest available information with the least number of tests [36]. The uniform design used in this paper can ensure the validity of the experimental results while reducing the workload. Thus, uniform design experiments were ideal for being introduced to investigate how process parameters affect uniformity. Five process parameters were determined by the preliminary reports [18,37,38,39]: melt temperature (X_1_), mold temperature (X_2_), injection speed (X_3_), injection pressure (X_4_), and holding time (X_5_). According to the recommendations and production experience offered by the polymer supplier, the other parameters were set as follows: holding pressure of 120 MPa and cooling time of 20 s. The levels and factors are listed in Table 2. The first five samples were discarded. After the process was stable, three parts were taken, and 45 parts were obtained under each cavity thickness. Specifically, the DPS was introduced to design uniform design experiments, as shown in Table 3.

### 2.3. Evaluation of Replication Uniformity

**Figure 3:**
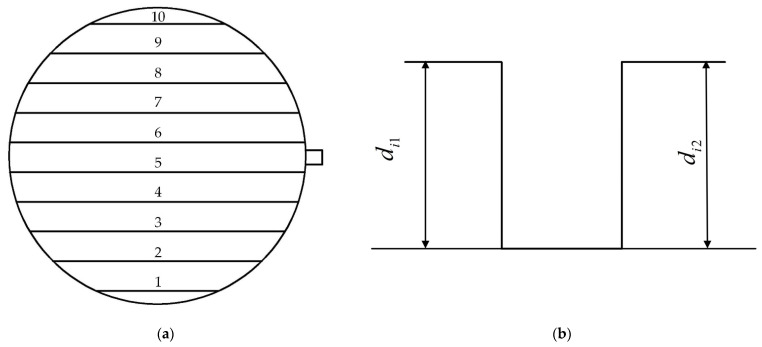
Schematic diagram of microstructure measurement: (**a**) microstructure measurement position; (**b**) methods for measuring microstructures.

An optical digital microscope (DSX1000, Olympus Co., Ltd., Tokyo, Japan) was utilized to obtain the 3-D micro-morphologies and 2-D cross-section profiles of the parts with appropriate magnifications. The measuring device has been calibrated by the technicians during installation, and the subsequent use process can ensure the accuracy and repeatability of the measurement. The measuring device has been calibrated by the technicians during installation, and the subsequent use process can ensure the accuracy and repeatability of the measurement. Ten of the microstructures at the same intervals along the diameter direction were selected and numbered from 1 to 10. The specific measurement method is shown in Figure 3b; relatively flat points are selected on the top and bottom of the rectangular microstructure. The *d*_*i*1_ and *d*_*i*2_ are measured, respectively, and the average value *d_i_* (*i* = 1, 2, …, 10) is taken. The replication depth of the microstructure array was chosen as the response variable for statistical analysis of the experimental result.

## 3. Results and Discussion

### 3.1. Quantification of Replication Uniformity

For a single part, uniformity is the consistency of the size of the microstructures at different locations. For different parts, it refers to the consistency of the size of the microstructures in the same position on the parts. We measured the depth of the microstructure to reflect the size of the microstructure. The uniformity quantification method is as follows.

For a single part, the uniformity of the microstructure $u_{i}$ at a certain position can be calculated by Equation (1) in this study,

(1) $$u_{i}=1-\frac{\left|d_{i}-\bar{d}\right|}{\bar{d}}$$

where *i* represents the number of microstructures selected on the plastic part, and $d_{i}$ is the depth of the rectangular microstructure. The $\bar{d}$ is the arithmetic mean of the microstructure depth, which can be calculated by Equation (2).

(2) $$\bar{d}=\frac{\sum_{i=1}^{10}d_{i}}{n}$$

The larger the $u_{i}$, the better the replication uniformity of the microstructure at this position. To compare the replication uniformity between parts, the standard deviation was introduced [10,17]. It was defined as Equation (3):

(3) $$\sigma_{Depth}=\sqrt{\sum_{i=1}^{n}{\left(d_{i}-\bar{d}\right)}^{2}/\left(n-1\right)}$$

where *n* is the total number of samples. The smaller the value of $\sigma_{\mathrm{Depth}}$, the smaller the depth difference between the various positions of the plastic part, which is better for the overall uniformity of the part.

The replication depth was used as the response variable. The standard deviation of the part was calculated by Equation (3) and the results are shown in Table 4.

When the molten polymer enters the mold cavities, the molten polymer in contact with the cavity wall quickly solidifies due to the relatively low temperature of the wall. The cooling rate for the molten polymer increases when the cavity temperature decreases [35]. Because the proportion of solidified plastic is relatively large compared to the macroscale cavity, short shots are frequently induced at cavities corresponding to microstructures [16]. Therefore, when the cavity thicknesses are 0.5 mm and 0.3 mm, there were one and five short shot groups, respectively, on the parts.

### 3.2. Effects of Cavity Thickness on the Replication Depth

**Figure 4:**
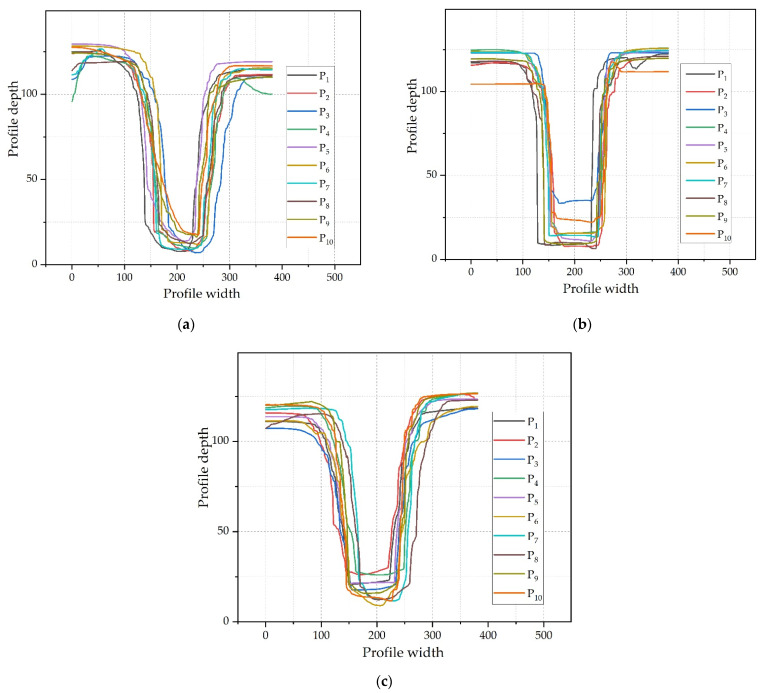
Microstructure profile: (**a**) cavity thickness is 0.3 mm; (**b**) cavity thickness is 0.5 mm; and (**c**) cavity thickness is 0.7 mm.

Figure 4 analyzes the influences of the cavity thickness on the microstructure replication profile. The P_i_ (i = 1, 2, …, 10) in Figure 4 represents the cross-section profiles of the 10 microstructures selected in Section 2.3. As shown in Figure 4, the microstructure profile is closer to the designed rectangle when the cavity thickness was 0.5 mm, while with cavity thicknesses of 0.3 mm and 0.7 mm, the grooves were round-shaped. Due to the presence of surface tension, the condensed layer cannot fully replicate the microstructure. The reason for good replication in the cavity thickness of 0.5 mm may be due to suitable cavity pressure and melt cooling rate during the injection. The replication profile at different positions fluctuates to different degrees because the flow state of the melt at each position is different.

**Figure 5:**
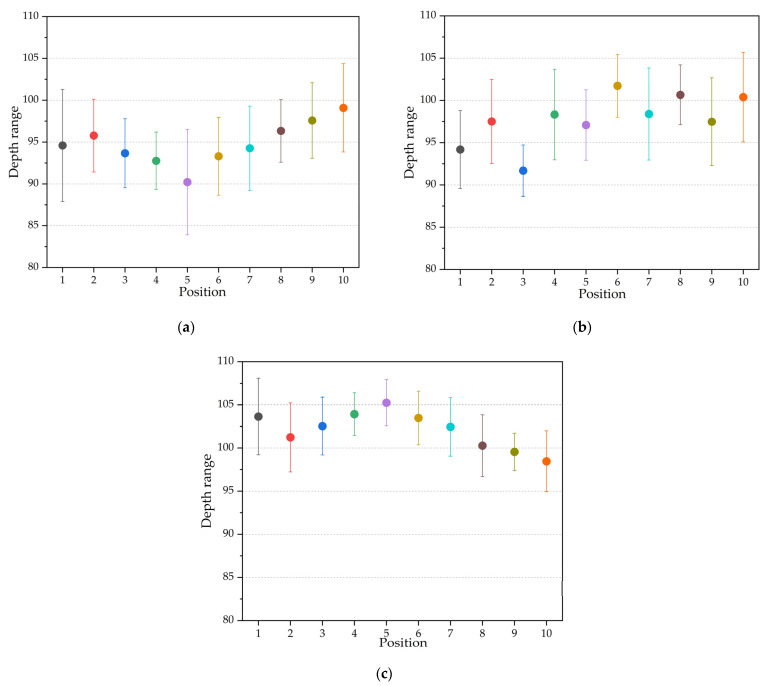
Replication depth: (**a**) the cavity thickness is 0.3 mm; (**b**) the cavity thickness is 0.5 mm; and (**c**) the cavity thickness is 0.7 mm.

Through the depth measured in Figure 3b, the influences of cavity thickness on the depth distribution are investigated, and the measured results are plotted in Figure 5.

By comparing the distribution of the microstructure replication depth, we can see that the replication depth of the parts with a cavity thickness of 0.7 mm is generally higher than the parts with a cavity thickness of either 0.3 mm or 0.5 mm. The results indicate that increasing the thickness of the cavity can promote replication depth. By comparing the fluctuation range of the replication depth at the same position under different thicknesses, we were able to ascertain that, with the increase in the cavity thickness, the influence of the process parameters on the replication depth of a single position is weakened.

### 3.3. Effects of Cavity Thickness on Replication Uniformity

#### 3.3.1. Effects of Cavity Thickness on Uniformity of Each Position

**Figure 6:**
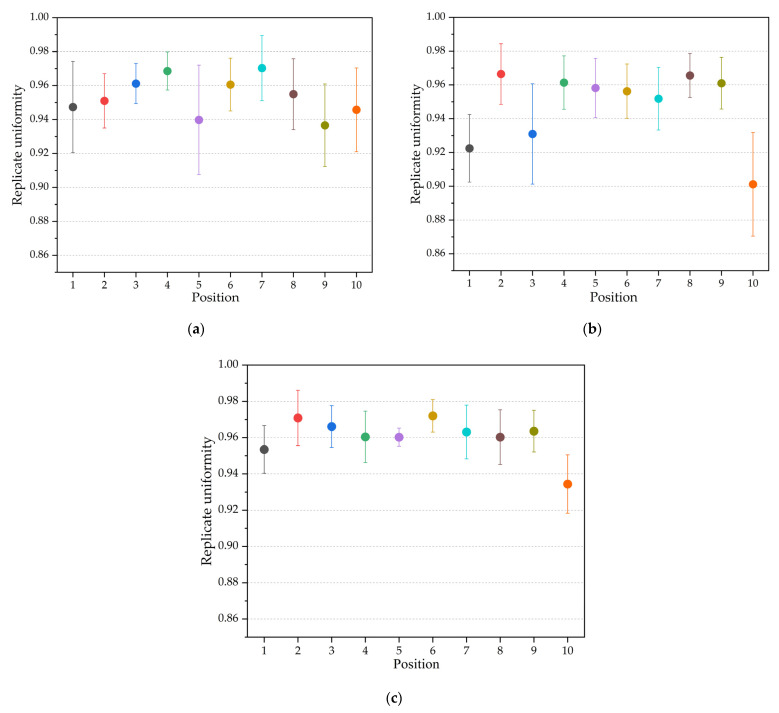
Replicate uniformity: (**a**) cavity thickness is 0.3 mm; (**b**) cavity thickness is 0.5 mm; and (**c**) cavity thickness is 0.7 mm.

To explore the replication uniformity of the microstructure depth, Figure 6 is plotted according to the result calculated by Equation (1).

Figure 6 exhibits the variation of the replication uniformity along the diameter. The abscissa axis represents the position of the measured microstructure and the vertical axis represents the replication uniformity. It can be seen that the uniformity for each position is mostly above 0.9, especially when the cavity thickness is 0.7 mm. When the cavity thickness is 0.7 mm, the fluctuation range of the replication uniformity at each position is significantly smaller than at the thicknesses of 0.3 mm and 0.5 mm, indicating that the increase in the cavity thickness can reduce the influence of the process parameters on the replication uniformity.

#### 3.3.2. Effects of Cavity Thickness on Overall Uniformity

**Figure 7:**
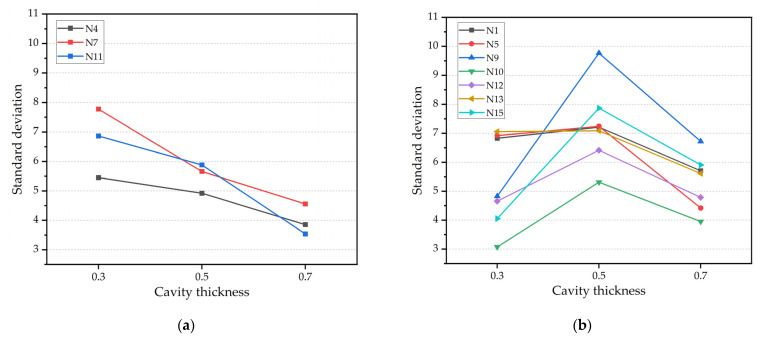
The relationship between cavity thickness and replication uniformity: (**a**) uniformity law under N4, N7, and N11; and (**b**) uniformity law under N1, N5, etc.

The overall uniformity of the part is quantified by Equation (3). To more intuitively analyze the effects of cavity thickness on overall uniformity, the results of three cavity thicknesses are summarized and drawn in Figure 7. The experimental results indicated that the effects of cavity thickness on overall uniformity can be divided into two situations depending on the process parameters. As displayed in Figure 7a, under these three sets of process combinations, the standard deviation decreases as the cavity thickness increases; that is, the part uniformity becomes better. As displayed in Figure 7b, the standard deviation increases first and then decreases as the cavity thickness increases, indicating that the replication uniformity first deteriorates and then becomes better. When the cavity thickness is changed from 0.5 mm to 0.7 mm, the standard deviation is reduced and the replication uniformity is improved under all process combinations. In general, as the cavity thickness increases, the overall uniformity improves. Because of the increased cavity thickness, the pressure distribution of the cavity is more uniform and the melt ensures better fluidity during the filling process. Among them, under the N11 combination, the standard deviation is significantly reduced by 39.82%. Indeed, the growth rate of cavity pressure is related to the pressure drop in the cavity, which is greater with a smaller thickness [30]. With the increase in cavity thickness, the pressure distribution became more uniform. In addition, uniform distribution of cavity pressure in the holding phase is achieved by increasing the cavity thickness [31]. When the cavity thickness is 0.3 mm, the main flow area is very thin and the scale effect leads to the flow state change, which makes the replication uniformity poor.

### 3.4. Effects of Process Parameters on Replication Uniformity

#### 3.4.1. Establish Mapping Model

According to previous research, when the important parameters were defined, the influences of the unimportant parameters on the uniformity could be ignored, including the holding pressure and cooling time. With the range of parameters, the regression equation between important parameters and uniformity is established by stepwise regression, which avoided omitting the important parameters. The regression equation is shown in Table 5. 

Additionally, the values of R^2^ and R^2^_adjust_ are 99.97% and 99.82%, respectively, which indicates that this model can explain the variable of replication uniformity well. Standard error of estimate S measures the difference between the estimated value estimated using the regression equation and the observed value of the standard deviation. The goodness of fit for this model is much higher than the goodness of fit reported in the literature with regression analyses [36,40,41]. Thus, the model accurately describes the relationship between the parameters and replication uniformity, and it is acceptable to discuss it in more detail based on the result of regression analysis.

#### 3.4.2. Influences of Process Parameters on Replication Uniformity

**Figure 8:**
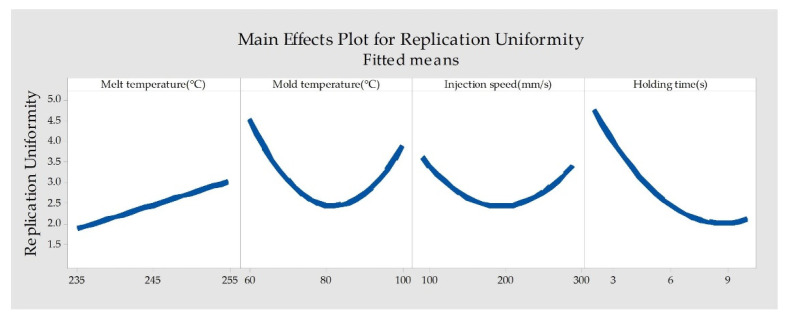
Main effects plot for replication uniformity.

We used a main effects plot, as shown in Figure 8, to examine the differences between level means for factors. Figure 8 shows that holding time is the most significant factor affecting uniformity, followed by mold temperature, melt temperature, and injection speed. The effect of holding time on the uniformity of the rectangular array was expected, since increasing the holding time allowed compensation for shrinkage before complete freezing, hence increasing its uniformity. The increased melt temperature was shown to be a source for decreasing uniformity of the microstructure array. This is because the higher melt temperature in the molding process, the filling end, and the gate position has significant temperature differences, so the uniformity of the plastic parts becomes worse. Moreover, as shown in Figure 8, with the increase in injection speed and mold temperature, the uniformity of the rectangular arrays first increases and then decrease.

## 4. Conclusions

The purpose of this study is to investigate the effects of cavity thickness and process parameters on the replication uniformity of rectangular microstructure arrays. The relationship between the replication uniformity of the rectangular arrays and the cavity thickness is studied through experiments. A stepwise regression analysis is introduced to establish the model of the relationship between process parameters and replication uniformity. The main conclusions are as follows:

- Increasing the thickness of the cavity can promote replication depth. With the increase in cavity thickness, the variation range of the replication depth at the same position under the influences of process parameters becomes smaller. The profile is closest to the designed rectangle when the cavity thickness is 0.5 mm, but the mean of the replication depth at 0.7 mm thickness is the best.
- There is a great correlation between cavity thickness and replication uniformity. With the increase in cavity thickness, the fluctuation in uniformity of each position caused by process parameters decreases. As the thickness of the cavity increases, the change law of the overall uniformity of the copy is divided into two types: one is a linear increase, the other first deteriorates and then improves, depending on the molding process parameters. In general, the cavity thickness of 0.7 mm provides the best replication uniformity. The replication uniformity is significantly increased by 39.82% from a cavity thickness of 0.5 mm to a cavity thickness of 0.7 mm.
- The process parameters have effects on the uniformity of the plastic parts with the microstructure arrays. Holding time is the most significant factor affecting uniformity, followed by mold temperature, melt temperature, and injection speed.

In general, the thickness of cavity and process parameters will greatly affect the replication uniformity. The experimental results provide important data for improving the replication uniformity of parts with microstructure arrays. In future work, sensors could be installed inside the cavity to interpret experimental phenomena.

## Figures and Tables

**Table 1:** Cavity thickness and block combination.

Cavity Thickness	Up Block	Down Block
0.3 mm	2.3 mm	6.9 mm
0.5 mm	2.5 mm	6.7 mm
0.7 mm	2.7 mm	6.5 mm

**Table 2:** Uniform table remarking U15 (32 × 53) in μIM.

Number	Factor X						
	X_1_ (°C)	X_2_ (°C)	X_3_ (mm/s)	X_4_ (MPa)	X_5_ (s)	X_6_ (MPa)	X_7_ (s)
1	235	60	50	90	2	120	20
2	245	80	110	120	4		
3	255	100	170	150	6		
4			230	180	8		
5			290	210	10		

**Table 3:** U_15_ (3^2^ × 5^3^) mixed level uniform table.

Factor	X_1_ (°C)	X_2_ (°C)	X_3_ (mm/s)	X_4_ (MPa)	X_5_ (s)
N1	245	60	90	210	6
N2	245	80	90	90	8
N3	245	100	230	120	10
N4	255	60	290	150	8
N5	235	100	170	210	8
N6	235	60	110	120	10
N7	245	80	170	150	2
N8	255	60	170	90	4
N9	235	60	230	180	2
N10	235	100	90	150	4
N11	255	100	230	180	6
N12	255	80	110	180	10
N13	255	100	110	120	2
N14	235	80	290	90	6
N15	245	80	290	210	4

**Table 4:** Experimental results for three cavity thicknesses.

RUN	Cavity Thickness 0.3 mm		Cavity Thickness 0.5 mm		Cavity Thickness 0.7 mm	
	Mean Depth	Standard Deviation	Mean Depth	Standard Deviation	Mean Depth	Standard Deviation
N1	85.61	6.82	98.92	7.21	102.53	5.71
N2			98.85	4.26	99.45	4.47
N3			101.15	7.35	106.16	2.43
N4	95.02	5.45	105.14	4.92	102.15	3.85
N5	101.07	6.92	100.31	7.24	97.49	4.42
N6					98.96	5.79
N7	94.18	7.78	92.11	5.66	106.64	4.56
N8			102.46	3.55	99.16	7.31
N9	105.39	4.83	105.09	9.77	101.31	6.72
N10	85.25	3.07	92.37	5.31	93.89	3.95
N11	97.00	6.86	96.71	5.89	106.55	3.54
N12	92.62	4.66	92.59	6.41	102.75	4.79
N13	88.20	7.06	90.97	7.09	101.74	5.61
N14			100.22	7.33	106.74	3.27
N15	103.16	4.05	103.13	7.87	105.72	5.91

**Table 5:** Regression model between process parameters and replication uniformity.

The Model Established in Minitab	R^2^	R^2^_adjusted_	S
Standard deviation= −107.12 + 0.5775 Melt temperature + 0.2335 Mold temperature + 0.1004 Injection velocity + 5.137 Holding time + 0.004411 Mold temperature × Mold temperature + 0.000109 injection velocity × injection velocity + 0.06121 Holding time × Holding time − 0.003998 Melt temperature × Mold temperature − 0.000407 Melt temperature × injection velocity − 0.021305 Melt temperature × Holding time + 0.003617Mold temperature × Holding time − 0.007142injection velocity × Holding time	99.97%	99.82%	5.61%

## Data Availability

Data share not applicable.
